# Noble-Metal based Metallic Glasses as Highly Catalytic Materials for Hydrogen Oxidation Reaction in Fuel Cells

**DOI:** 10.1038/s41598-019-48582-7

**Published:** 2019-08-20

**Authors:** Vahid Hasannaeimi, Sundeep Mukherjee

**Affiliations:** Department of Materials Science and Engineering, University of North Texas, Denton, Texas 76203 USA

**Keywords:** Fuel cells, Electrocatalysis

## Abstract

Electro-catalyst design with superior performance and reduced precious metal content (compared to state-of-the-art Pt/C) has been a challenge in proton exchange membrane fuel cells, preventing their widespread adoption. Metallic glasses have recently shown promising performance and large electrochemical surface area in catalytic reactions. The electro-catalytic behavior of recently developed Pt-, Pd-, and Pt/Pd-based metallic glasses was evaluated in this study using scanning electrochemical microscopy. The influence of chemistry and electronic structure on catalytic behavior was studied using scanning kelvin probe technique. The work function for the metallic glasses was lower by 75 mV to 175 mV compared to pure Pt. This resulted in higher catalytic activity for the amorphous alloys, which was attributed to the ease of charge transfer on the surface. The binding energy for the metallic glasses, measured using X-ray photoelectron spectroscopy, was higher by 0.2 eV to 0.4 eV. This explained easier removal of adsorbed species from the surface of amorphous alloys. The synergistic effect of Pt and Pd in alloys containing both the noble metals was demonstrated towards hydrogen oxidation reaction.

## Introduction

Fuel cells offer very high energy density among energy storage/conversion devices and are classified primarily based on their electrolyte or operation temperature^1^. Proton exchange membrane fuel cells (PEMFCs) are particularly attractive because they use hydrogen as fuel and operate at low temperatures (50 to 100 °C). PEMFCs have emerged as a promising power source for transportation and portable energy storage and are likely to replace aging alkaline fuel-cell technology used in space shuttles^2^. However, one of the major bottlenecks in widespread use of PEMFCs has been the high cost of platinum group metals (PGM) used as catalysts. Pt- and Pd-based alloys including Pt-Ru^3^, Pt-Sn^4^, Pt-Pd^5^, Pd-Co^6^, Pd-Au^7^, Pt-Ru-Mo^8^ and Pt-Ru-Ni^9^ have shown significantly higher electro-catalytic activity in both acidic and alkaline media compared to pure noble metal catalysts, allowing for reduced PGM loading. This has been attributed to the reduction in binding energy when the noble metals are alloyed with more oxophilic elements. Nevertheless, it should be noted that either too strong or too weak M-H (M stands for metal surface) binding energy may result in poor catalytic behavior^10^. Furthermore, there is limited understanding and open questions related to the mechanism of oxidation/reduction and its relationship with electronic characteristics such as binding energy and work function (WF)^11^. According to the free energy of activation (ΔG*) for proton (H^+^) reduction, work function maybe directly correlated with the strength of M-H bond. However, the reported trends are inconsistent or even contradictory^12,13^.

 A major concern in PEMFCs is the degradation and dissolution of catalysts and carbon support under fuel cell operating conditions^14^, which determines a fuel cell performance in addition to catalyst chemistry. The majority of investigations on electrocatalysts have been limited to crystalline materials^5,15,16^. Only recently, bulk metallic glasses (BMGs) have attracted attention as highly active electro-catalysts owing to the high density of low-coordination sites on their surface, metastable amorphous structure, and high durability^17–20^. However, the effect of different amorphous chemistries on catalytic activity is not well understood and has not been reported. Catalytic reactions mainly consist of diffusion, adsorption, and desorption of chemical species along with charge transfer process. Work function (WF), which is the minimum energy required for removing an electron from a material’s surface, plays a key role in the charge transfer process and shown to be correlated with catalytic activity^21,22^. Scanning kelvin probe (SKP) may be used for measuring the contact potential difference (CPD) to generate high-resolution maps of work function or potential distribution^23,24^. Metal-catalyzed reaction rates depend exponentially on catalyst work function and referred to as electrochemical promotion of heterogeneously catalyzed (EPOC) reactions^25^. Furthermore, an inverse correlation between electrocatalytic behavior of polycrystalline Pt-based nanoparticles with the work function has been recently shown for oxygen reduction^26^. Combining SKP with *in-situ* electroanalytical techniques such as scanning electrochemical microscopy (SECM)^27^, may provide valuable insights into the underlying correlation between electronic and electro-catalytic characteristics of recently developed metallic glasses. Here, we report on the electro-catalytic activity of a series of Pd- and Pt-based metallic glasses using SECM and SKP for hydrogen oxidation reaction (HOR). SKP analysis was utilized for fundamental understanding of the role of electronic structure on electrochemical reactivity of the amorphous alloys. Synergistic effects of Pt/Pd toward HOR were shown for alloys containing both the noble metals.

## Results and Discussion

**Figure 1:**
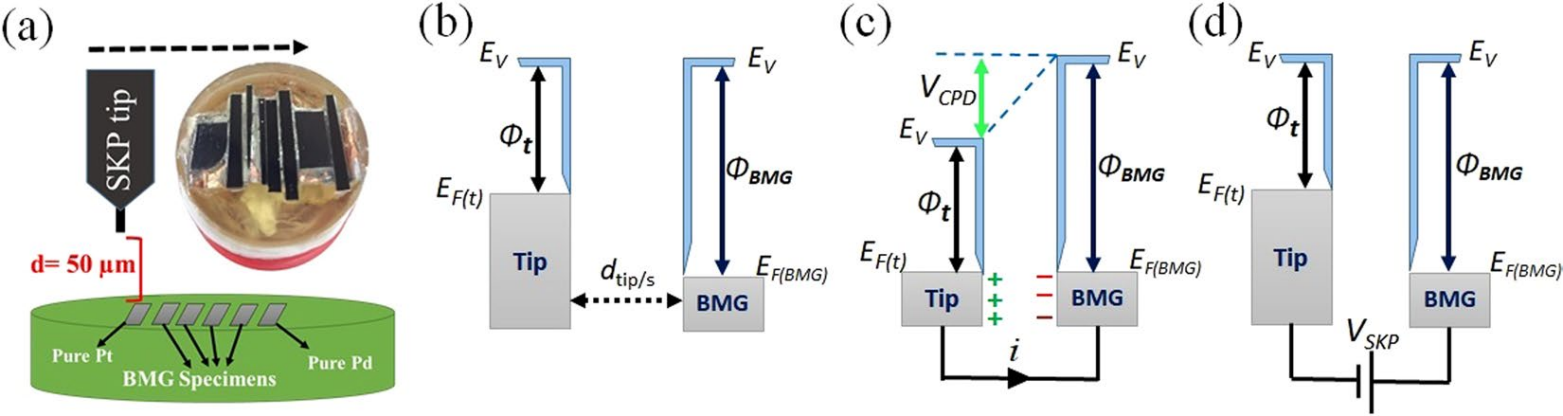
(**a**) The experimental setup and a schematic showing the array of amorphous alloys and pure Pt and Pd mounted side by side in a non-conductive resin. Energy level diagram of tip and substrate (BMG) when: (**b**) there is no electrical contact between the tip and substrate; (**c**) tip and the substrate are connected electrically while a contact potential difference (V_CPD_) is generated; and (**d**) an external electrical bias is applied to nullify V_CPD_, which is the SKP potential (V_skp_). E_v_ is the vacuum energy level, and E_F(t)_ and E_F(BMG)_ are Fermi levels of the tip and the BMG, respectively.

The experimental setup and a schematic showing the array of amorphous alloys and pure Pt and Pd mounted side by side in a non-conductive resin are shown in Fig. 1(a). The tip was moved laterally over the surface of the specimen at a fixed distance of 50 µm from the surface. Energy diagrams of the tungsten tip and BMG substrate separated at a certain distance (*d*_*tip/s*_) are shown schematically in Fig. 1(b). The vacuum levels are the same while the Fermi levels are different for each material^28^. When the tip and BMG sample are connected electrically, electron flow results in Fermi energy alignment and the system reaches equilibrium. However, a contact potential difference (V_CPD_) is formed between the tip and sample as shown in Fig. 1(c). The external opposite potential (V_SKP_) that nullifies V_CPD_ (Fig. 1(d)) is proportional to the relative work function difference between the tungsten tip and BMG sample (V_SKP_ ~ Ф_BMG_-Ф_tip_, in which Φ represents the work function).

**Figure 2:**
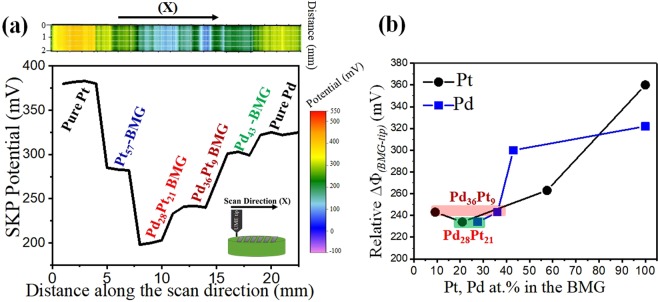
(**a**) SKP map/line scan of the BMGs along with pure Pt and Pd measured over an area of 2 mm × 25 mm with a tungsten micro tip and the inset showing tip scanning direction over the array of amorphous alloys; (**b**) Relative work function difference of the BMGs as a function of Pt and Pd content with alloys containing both Pd and Pt marked with two separate boxes.

SKP analysis was performed with a constant tip to specimen distance of 50 µm. The potential map and a single line scan of V_SKP_ are shown in the Fig. 2(a). Positive values for the BMGs indicate their higher work function compared to tungsten $({{\rm{V}}}_{{\rm{CPD}}}=\frac{{{\Phi }}_{sample}-{{\Phi }}_{tip}}{-e})$. Pure Pt showed the highest work function followed by pure Pd and the amorphous alloys. The BMGs with higher fraction of noble metal showed higher work function. A roughly linear correlation between composition and work function has been reported for binary alloys of AgAu^29^, PtRh and NiCu^30^, showing an increasing trend with increase in the fraction of higher work function element. A similar trend was observed for the BMGs, as shown in Fig. 2(b). The alloys containing both Pt and Pd showed the lowest work function values, ~150 mV lower SKP potential compared to pure Pt. The rate of metal-catalyzed reactions in solid oxide fuel cells was shown to depend exponentially on average catalyst WF over a wide range of work functions (0.3–1 eV)^31^. In the present study, WF for all BMGs was found to be lower compared to pure Pd and Pt. Higher electronic conductivity results in higher reaction rates over the catalyst surface due to easier electron transfer^32^. It has been reported that the work function of contaminated Ni films decreased noticeably with hydrogen adsorption on its surface which resulted in increase of conductivity^33^. Similarly, the ability of Pt-based nanoparticle catalysts to activate molecular oxygen has recently been shown to have an inverse correlation with WF^26^. As mentioned in the Materials and Methods section, surface roughness does not affect the work function values from SKP measurement because of surface topography correction. A high resolution topography history of the samples was initially recorded using constant height mode of the SKP microscope. The results were calibrated using the topography history to ensure that they were attributed only to the work function difference between the tip and the sample. The average surface roughness (R_a_) was calculated using Scanning Probe Microscopy to be 322.5 ± 20.5 nm for all specimens.

**Figure 3:**
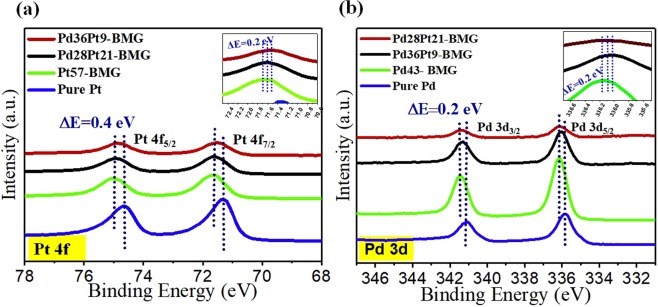
XPS spectra of (**a**) Pt 4f and (**b**) Pd 3d for the BMGs and pure Pt and Pd with the insets showing close-up view for peak positions in the amorphous alloys.

To determine the possible correlation between lower work function of BMGs and their binding energies, the chemical valence state of the samples were obtained using XPS. The recorded Pt 4 f and Pd 3d core levels are shown in Fig. 3. The core levels for Pt and Pd are separated into Pt 4f_7/2_, Pt 4f_5/2,_ Pd 3d_5/2_, and Pd 3d_3/2_, respectively, due to spin orbital splitting. The binding energy (BE) values at 71.3 eV and 74.6 eV correspond to Pt 4f_7/2_ and Pt 4f_5/2_, respectively, which are attributed to Pt°. The absence of other Pt valence states such as Pt^2+^ indicates that Pt is not present in the form of oxide or hydroxide on the surface, but in its zero-valent state. Interestingly, the BE of Pt for the Pt_57_-BMG showed a positive shift of + 0.4 eV with respect to pure Pt. The change in BE of Pt in the BMGs is likely related to the difference in electronegativity of the alloying elements (Cu, Ni and P). Moreover, it has been shown that the shift in BE is typically correlated to the change in *d*-band center, Fermi energy, and work function^34^. The lower work function for the BMGs correspond to the positive shift of Pt bond strength in these metallic glasses. The upshift of bond strength in Pd- and Pt-based metallic glasses is indicative of a downshift in *d*-band center^34^. Any shift in *d*-band center for the Pt surface would result in a surface core-level shift in the same direction^35^. The reduction in density of states at the Fermi level due to electron transfer from less electronegative elements to Pt or Pd has been reported to reduce the bond strength of Pt and adsorbed chemical species, leading to improved electro-catalytic activity of Pt-M (M = Ru, Sn) alloys^36^. In the present study, the chemisorption of protons on the Pd- and Pt-based amorphous alloy surface would be weakened since a higher *d*-band center corresponds to weaker M-H bonding^37^. Majority of the Pt is present in zero-valent state for all the BMG specimens. This may result in enhanced catalytic activity as zero-valent Pt provides more active sites^38^. A small difference (~0.2 eV) in Pt BE is seen among the different BMG compositions (the inset of Fig. 3(a)). A similar trend for Pd_43_-BMG was observed with respect to pure Pd. The BE values at 335.8 eV and 341.1 eV correspond to Pd 3d_5/2_ and Pd 3d_3/2_, respectively, which are related to Pd°. A positive shift of +0.4 eV was observed for Pd° in the Pd_43_-BMG. Higher BE of Pt and Pd in the Pt- and Pd-based BMGs may result in weakened chemisorption during catalysis. The binding energies of Pt and Pd for different BMG catalysts are summarized in Table 1. The BMGs showed higher binding energy of Pt and Pd in addition to reduced work function.

**Table 1:** Binding energies of Pt and Pd for the different metallic glass catalysts.

Catalysts	Binding energy (eV) Pt 4f_7/2_ 4f_5/2_		Pd 3d_5/2_ 3d_3/2_		ΔE (eV) (difference in binding energy vs. pure Pt/Pd)
Pure Pt	71.3	74.6	—	—	—
Pt_57_-BMG	71.7	75	—	—	+0.4
Pd_28_Pt_21_-BMG	71.6	74.9	336.1	341.4	+0.3
Pd_36_Pt_9_-BMG	71.5	74.8	336	341.3	+0.2
Pd_43_-BMG	—	—	336.2	341.5	+0.4
Pure Pd	—	—	335.8	341.1	—

**Figure 4:**
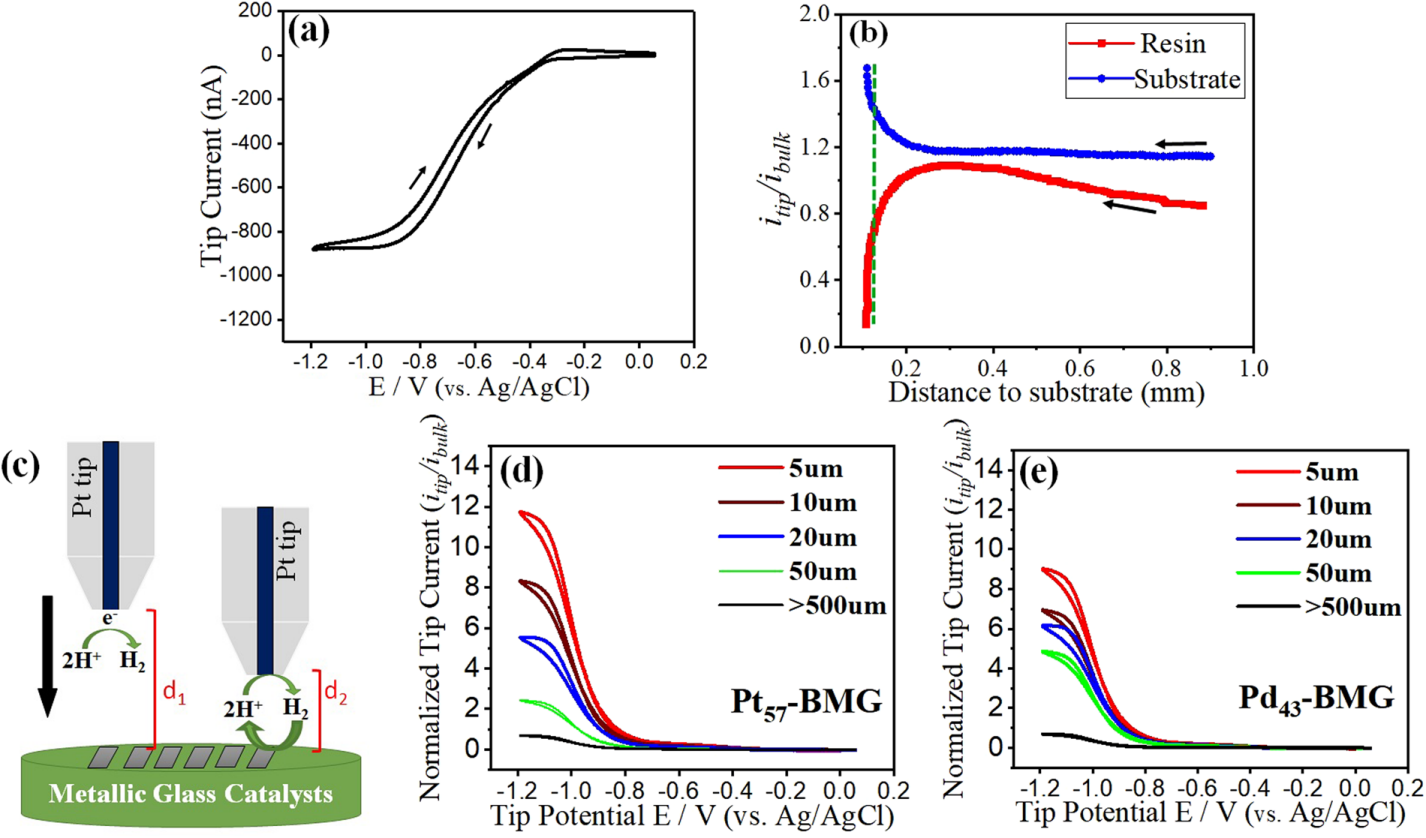
(**a**) Cyclic voltammogram of Pt microelectrode in 0.01 M H_2_SO_4_ + 0.1 M Na_2_SO_4_ solution at a scan rate of 50 mV/s in bulk solution (d >500 µm); (**b**) Z-approach curves of Pt UME over Pt_57_-BMG and non-conductive resin, the dashed line shows the selected tip/substrate distance; (**c**) Schematic of the response of BMG substrate/SECM tip at various d values, at d_1_ the tip/substrate separation is too high for getting feedback from the substrate, while at d_2_ the substrate is close enough to the tip to oxidize protons; (**d**) Tip current normalized by the bulk value in 0.01 M H_2_SO_4_ + 0.1 M Na_2_SO_4_ solution at various tip-substrate distances over Pt_57_-BMG. The tip potential was scanned between −1.2 V and 0.1 V (vs Ag/AgCl) at 100 mV/s, while the substrate was at OCP; (**e**) Tip current normalized by the bulk value in 0.01 M H_2_SO_4_ + 0.1 M Na_2_SO_4_ solution at various tip-substrate distances over Pd_43_-BMG.

To investigate the electro-catalytic activity of the amorphous alloys with respect to pure noble metals, SECM imaging was employed in an electrolyte of 0.01 M H_2_SO_4_ mixed with argon saturated 0.1 M Na_2_SO_4_. Hydrogen oxidation reaction was studied as it is the primary anodic reaction in PEMFC. Cyclic voltammetry at the Pt UME was recorded in bulk solution in the potential range of −1.2 V to +0.1 V (vs Ag/AgCl). At potentials higher than −0.4 V, the tip current approached zero (Fig. 4(a)). Since, hydrogen evolution reaction (HER) potential is roughly around −0.25 V^39^, hydrogen would be oxidized in this potential range (*V* > −0.4 *V*). Due to large over-potential of Pt tip for HER, significant reduction of proton was not observed until the potential reached values below −0.8 V. The tip current reached a steady state in the potential range of −0.9 V to −1.2 V (vs. Ag/AgCl), with mass transport current reaching a value of −900 nA. Further negative tip potentials (*V* < −1.2*V*) led to the reduction of bulk water followed by formation of H_2_ bubbles. Approach curves were measured at various tip/substrate separations over the electrically conductive substrate and neighboring non-conductive resin. The tip and substrate were held fixed at −1.2 V and open circuit potential (OCP), respectively. The measured current was normalized with respect to the tip current measured in bulk solution. A positive feedback was seen at the UME tip (Fig. 4(b)) and the i_tip_/i_bulk_ increased as the tip approached the BMG surface due to catalytic oxidation of locally generated hydrogen (H_2_). In contrast, a negative feedback was observed when the Pt tip approached the nonconductive resin surface. The reactivity of BMGs towards HOR was imaged by performing cyclic voltammetry. Depending on the tip/substrate distance, the samples showed different feedback towards HOR (Fig. 4(c)). At large tip/sample separation (shown schematically as *d*_1_ in Fig. 4(c)), proton reduction occurred at the tip and there was no feedback from the substrate. With reduction of tip-substrate separation (shown schematically as *d*_2_ in Fig. 4(c)), hydrogen oxidation from Pt_57_-BMG surface resulted in positive feedback. Hydrogen oxidation enhanced proton concentration in the vicinity of the Pt UME and increased the measured current at the tip as shown in Fig. 4(d). The recorded tip currents were normalized with respect to the measured values in bulk solution (Fig. 4(d)). A uniform tip response was observed when the tip was far from the substrate (*d* > 500 μm), with a nearly fixed current at the bulk diffusion-limited value $(\frac{{i}_{tip}}{{i}_{bulk}}=1)$. At this separation, no feedback from the substrate was observed. The tip current significantly increased as the tip/substrate separation was reduced due to positive feedback from the BMG surface. Maximum tip current was recorded when the tip/substrate distance was ~5 µm. About twelve-fold increase in the tip current was observed for Pt_57_-BMG at 5 µm tip/sample separation as compared with bulk solution. The increase in tip current response was similar for Pd_43_-BMG, but the magnitude was lower (nine-fold increase in this case as shown in Fig. 4(e)). Although the electronic properties of Pd and Pt are comparable, hydrogen adsorption is stronger on Pd as compared with Pt, resulting in lower catalytic currents for Pd and its alloys^10^. The cyclic voltammetry plots for the other amorphous alloys were similar and are not shown.

**Figure 5:**
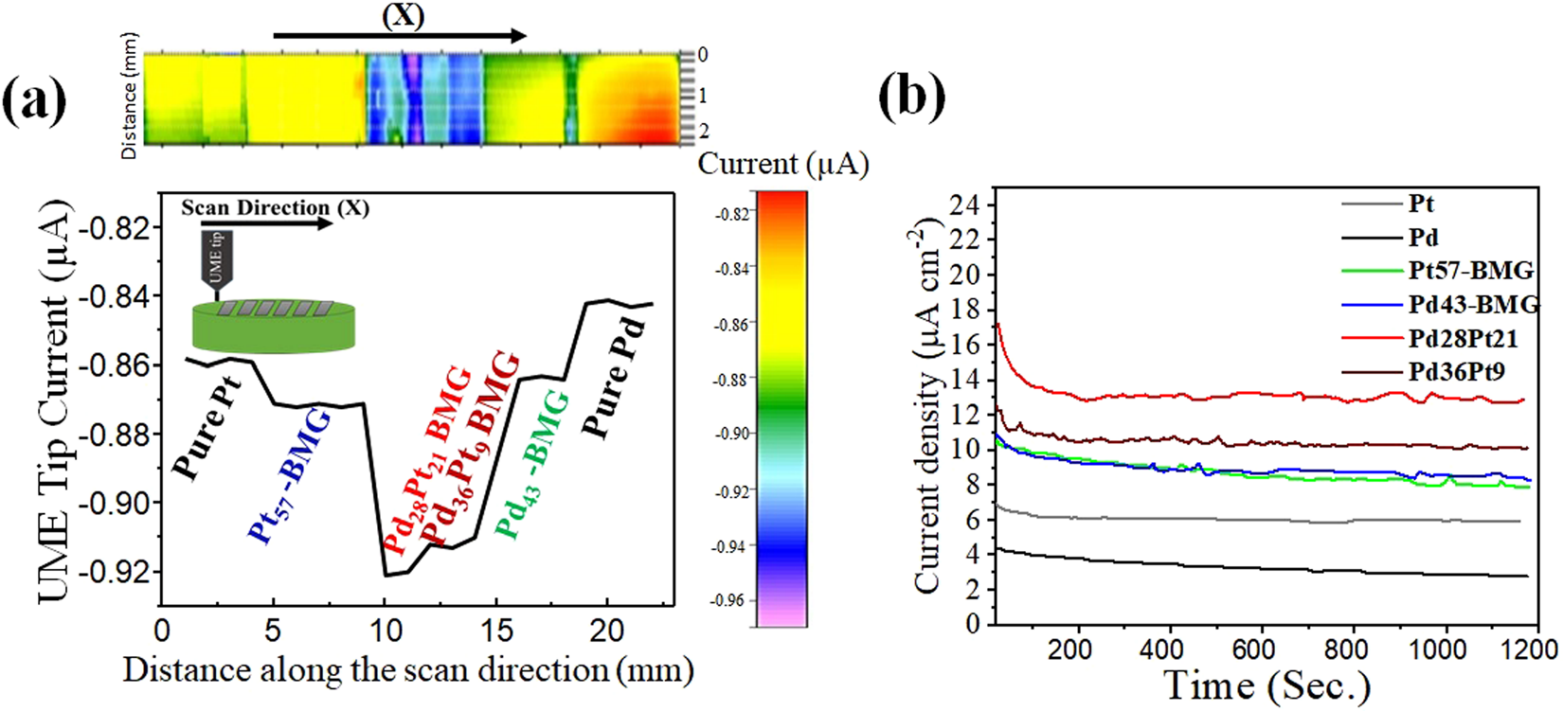
(**a**) Pt UME reduction current distribution map along with a single line scan over the BMGs in 0.01 M H_2_SO_4_ + 0.1 M Na_2_SO_4_ solution at a fixed tip/substrate distance of 5 µm, the tip potential was held fixed at −1.2 V (vs. Ag/AgCl), while the sample was at OCP; the inset shows the tip scanning direction over the array of amorphous alloys; (**b**) Chronoamperometric plots of the metallic glass catalysts and pure Pt and Pd at a potential of −1100 mV (vs Ag/AgCl) in 0.01 M H_2_SO_4_ + 0.1 M Na_2_SO_4_.

To measure the kinetics of catalytic reaction, the Pt tip polarized at −1.2 V was scanned over all the alloys at a fixed tip/substrate separation of 5 µm. No over-potential was applied to the catalysts during the experiments. The variation in Pt tip current is proportional to the catalytic rate constant of the amorphous alloys. More negative values correspond to higher reactivity of the substrate. The plotted SECM map and the corresponding Pt tip current line scan are shown in Fig. 5(a). The variation in UME tip current was solely from the difference in chemistry of the BMGs since all the alloys were fully amorphous and had the same surface finish. Pure Pt and Pd specimens showed lower catalytic reactivity towards HOR compared to the amorphous alloys. Both the Pd_43_- and Pt_57_-BMGs exhibited higher activity compared to their pure counterparts, with the UME tip reduction current increase of 30 nA and 20 nA, respectively. The amorphous alloys containing both Pd and Pt exhibited the highest activity towards HOR among the systems studied. Higher specific activity (kinetic current per unit surface area of a catalyst) has recently been shown for Pd-Pt nano-dendrites towards ORR, which was related to the preferential exposure of more active crystalline facets (Pt(111) is more favored in the presence of Pd) on the Pt branches as compared with small Pt nanoparticles in Pt/C^40^. This synergistic effect has been attributed to the modification of Pt *d*-band center and weakening of electronic interaction between Pt atoms and oxygenated species. Most of the reports on synergistic effects of Pt/Pd towards different catalytic reactions have been attributed to crystallographic modification of Pt. However, BMGs have a homogenous amorphous structure without any crystalline facets^41^. The high density of low coordination sites in the amorphous alloys likely resulted in enhanced surface reations and higher catalytic activity^42^.

In order to evaluate the stability of the amorphous catalysts towards HOR, chronoamperometry was performed for all the samples. All the alloys reached steady-state current densities within ~200 sec (Fig. 5(b)). However, the current densities for the amorphous alloys containing both Pd and Pt were significantly higher compared to others, which may be attributed to the synergistic effect between these elements. The trend in chronoamperometry results were along the same lines as the SECM activity map. Modification of electronic structure may account for the better catalytic performance of Pt/Pd-based BMGs. Work function values for BMGs containing both Pd and Pt were lower compared with Pd_43_- and Pt_57_-based BMGs, promoting easier electron transfer. This also explains the shift in binding energies to higher values reducing the chemisorption of absorbed species for Pt/Pd alloys^34^.

## Conclusions

Scanning electrochemical microscopy was utilized to evaluate the catalytic performance of several Pd- and Pt-based amorphous alloys towards hydrogen oxidation reaction, the main anodic reaction in PEM fuel cells. The metallic glasses showed higher catalytic activity compared to pure Pt and Pd, which was attributed to their relatively lower work function measured by scanning kelvin probe (SKP). The work function for the metallic glasses was lower by 75 mV to 175 mV compared to pure Pt. This resulted in higher catalytic activity for the amorphous alloys, which was attributed to the ease of charge transfer on the surface. The shift in Pt and Pd binding energies to higher values, as shown by XPS, supported the weakened chemisorption of adsorbed species and more enhanced catalysis. The alloys containing both Pt and Pd showed the highest catalytic activity towards HOR, attributed to the synergy between the two noble elements by electronic structure modification.

## Materials and Methods

### Materials

In this study, four different metallic glass systems were chosen: one Pt-based bulk metallic glass of composition Pt_57.5_Cu_14.7_Ni_5.3_P_22.5_^43^ (referred to as Pt_57_-BMG), one Pd-based BMG of composition Pd_43_Ni_10_Cu_27_P_20_^44^ (referred to as Pd_43_-BMG), and two recently developed BMGs containing both Pt and Pd, namely Pd_27.5_Pt_20.9_Cu_22.6_Ni_8.2_P_20.9_ (referred to as Pd_28_Pt_21_ BMG) and Pd_36.1_Pt_9.2_Cu_25.1_Ni_9.2_P_20.3_ (referred to as Pd_36_Pt_9_ BMG). All the alloys were prepared in the form of amorphous rods by induction melting high purity starting elements in vacuum-sealed quartz tubes followed by water quenching molten samples after appropriate B_2_O_3_ fluxing. The glass transition (T_g_) and crystallization (T_x_) temperatures, measured using differential scanning calorimetry (DSC-Netzsch) with a heating rate of 20 K/min, are summarized in Table 2.

**Table 2:** The composition, glass transition temperature (T_g_), crystallization temperature (T_x_), and the difference between glass transition and crystallization temperatures (ΔT) for the BMGs.

BMG Alloy	Composition	T_g_ (°C)	T_*x*_ (°C)	ΔT (*T*_*x*_ *− T*_*g*_)
Pt_57_-BMG	Pt_57.5_Cu_14.7_Ni_5.3_P_22.5_	235	315	80
Pd_28_Pt_21_ BMG	Pd_27.5_Pt_20.9_Cu_22.6_Ni_8.2_P_20.9_	265	350	85
Pd_36_Pt_9_ BMG	Pd_36.1_Pt_9.2_Cu_25.1_Ni_9.2_P_20.3_	310	380	70
Pd_43_-BMG	Pd_43_Ni_10_Cu_27_P_20_	310	390	80

Pure crystalline Pd and Pt were used as reference catalysts. To analyze the surface chemical valence states of the BMG specimens, X-ray photoelectron spectroscopy (XPS-PHI 5000 Versaprobe) was done. The BMGs were then mounted in a hard resin side by side, with a conductive copper tape on the other side for electrochemical studies. The mounted specimens were polished to a 0.1 µm surface finish followed by Vibromet polishing for 24 h in 0.04 µm colloidal silica suspension. To remove any contamination from the surface, the samples were cleaned ultrasonically in acetone for 10 min followed by isopropanol for 15 min and subsequently washed with distilled water. The roughness of the surface was measured using high resolution scanning probe microscopy (SPM).

### Electrochemical measurements

To measure relative work function of the specimens, SKP analysis was performed in dry lab air. A pure tungsten microprobe (150 µm in diameter) was used as reference electrode vibrating at a frequency of 80 Hz with an amplitude of 30 µm normal to the surface. A backing potential was applied to minimize the capacitance between probe and working electrode. A constant tip to substrate distance of 50 µm was used for the experiment. A high resolution topography history of the samples was initially recorded using constant height mode (CHM) of the SKP microscope (VersaSCAN-Princeton Applied Research) with step size of 20 µm over an area of 2 mm × 25 mm. The SKP potential map was recorded over all the samples with a fixed step size, potential gain, time constant and sensitivity. Finally, the results were calibrated using the topography history to ensure that they were attributed only to the work function difference between the tip and the amorphous alloys.

For measuring the electrocatalytic activity of the BMGs, high resolution SECM (VersaSCAN-AMETEK) was used in redox competition mode. The key components of the system included a potentiostat (VersaSTAT 3 F) coupled with a positioning system, an ultramicroelectrode (UME) and BMG sample as the working electrode. The tip/sample separation was optimized at 5 µm, and kept constant for all catalytic reaction studies using software based tilt correction. The catalytic performance measurement was done over an area of 2 mm × 25 mm, with step size of 10 µm at a fixed tip/substrate separation of 5 µm. Ag/AgCl/3 M KCl (E = 0.196 V vs SHE) and a Pt wire were used as the reference and counter electrodes, respectively. A Pt UME with a radius of 10 µm encased in borosilicate glass tube was used as SECM tip. The RG ratio (where RG is the ratio of insulating sheath radius divided by the Pt tip radius) of the probe was ~10 with a disk-in-plane geometry. The UME was polished gently with Alumina slurry, carefully washed with distilled water, and pre-treated with potential cycling in 0.1 M H_2_SO_4_ before use. All cyclic voltammetry experiments were performed at a potential sweep rate of 50 mVs^−1^ and room temperature.

For determining the optimum distance between the tip and substrate, approach curves were plotted using Pt UME as the working electrode, while the insulator resin and conductive BMG alloys were employed as substrates. The distance at which the tip current changed to 75% of bulk solution current was chosen as the desired tip/substrate separation. All electrochemical experiments were conducted in aqueous solution containing H_2_SO_4_ and Na_2_SO_4_, deaerated with argon gas prior to measurement. To investigate the stability of the alloys towards HOR, chronoamperometry was performed in a three electrode cell set up with Pt wire and Ag/AgCl electrodes as counter and reference, respectively, at a scan rate of 10 mV/s for 1200 sec. The measured current was normalized by the surface area of the specimens.

## Data Availability

All data generated or analysed during this study are included in this published article.
